# Prognostic value of lipid profiles after radical prostatectomy: a systematic review and meta-analysis

**DOI:** 10.1186/s12944-019-1068-6

**Published:** 2019-05-28

**Authors:** Xiaonan Zheng, Xin Han, Hang Xu, Jianzhong Ai, Lu Yang, Qiang Wei

**Affiliations:** 1 Department of Urology, Institute of Urology, West China Hospital, Sichuan University, Chengdu, Sichuan People’s Republic of China; 2 West China Medical School, Sichuan University, Chengdu, Sichuan People’s Republic of China

**Keywords:** Lipid profiles, Prognostic value, Radical prostatectomy, Advanced pathologic tumor features, Biochemical recurrence.

## Abstract

**Background:**

Lipid profiles are believed to play an important role in the tumorigenesis and progression of prostate cancer (PCa), but research combining those data is lacking. Therefore, this meta-analysis aims to assess the prognostic role of lipid profiles after RP.

**Method:**

We systematically searched PubMed, Embase, and Cochrane Library Central Register of Controlled Trials for articles evaluating association between lipid profiles and prognosis after RP. Odds ratio (OR) and hazard ratio (HR) of lipid profiles for advanced pathological tumor features and biochemical recurrence (BCR) were extracted and pooled OR and HR were calculated. Newcastle-Ottawa scale was used for study quality assessment and funnel plot was used for evaluating publication bias.

**Results:**

Twelve articles involving 11,108 patients were eventually selected. We found low HDL was associated with more frequent occurrence of pathological T stage (pT) ≥ T3 (pooled OR = 1.29, 95% CI 1.07–1.56) and Gleason score (GS) ≥8 (pooled OR = 1.32, 95% CI 1.02–1.72) after RP. Hypertriglyceridemia was also linked with higher risk of pT ≥ T3 (pooled OR = 1.20, 95% CI 1.01–1.42) and positive surgical margin (PSM) (pooled OR = 1.36, 95% CI 1.11–1.65). However, no significant association was observed between BCR and abnormal lipid profile levels.

**Conclusion:**

Low HDL level was associated with more common occurrence of pT ≥ T3 and GS ≥8, and elevated triglycerides level was linked higher risk of pT ≥ T3 and PSM, but none of the lipid subfractions was correlated with biochemical recurrence after RP.

**Electronic supplementary material:**

The online version of this article (10.1186/s12944-019-1068-6) contains supplementary material, which is available to authorized users.

## Introduction

Prostate cancer is (PCa) one of the most commonly diagnosed cancer in men worldwide [1]. For localized PCa, radical prostatectomy (RP) has been strongly recommended as a standard treatment option [2, 3]. However, biochemical recurrence (BCR) and aggressive clinicopathological features are not rare after RP [4].

Cholesterol has been confirmed as promising biomarkers of cardiovascular disease [5]. Multiple studies have also investigated the role of serum lipid profiles, including total cholesterol (TC), low-density lipoprotein (LDL), high-density lipoprotein (HDL) and triglycerides (TG), in the incidence of PCa and generated conflicting results [6–9]. A meta-analysis integrated those outcomes and suggested that serum lipid profiles are not associated with PCa risk [10]. Similarly, the prognostic value of lipid profiles regarding BCR and postoperative aggressive clinicopathological features after RP remains controversial [11–14], but yet no research has combined those data and clearly clarified the prognostic role of lipid profiles after RP.

Therefore, based on those disputed studies, the purpose of the current meta-analysis is to comprehensively evaluate the association between serum lipid profiles with BCR and aggressive clinicopathological features after RP.

## Method

### Search strategy and study selection

PRISMA guidelines were followed to perform this systematic review and meta-analysis [15, 16]. We systematically searched PubMed, Embase, and Cochrane Library Central Register of Controlled Trials to date using terms including “radical prostatectomy”, “lipid”, “cholesterol”, “high density lipoprotein”, “low density lipoprotein”. “metabolic syndrome”, which may comprise LDL and TG, was also one of our searching terms. The references cited by the finally selected articles were also reviewed.

Studies assessing the association between outcomes post RP and lipid profiles were potentially eligible for inclusion. The detailed inclusion criteria were as following: 1. Patients must be treated with only RP, no radiation therapy or chemotherapy was administrated alongside; 2. Study must evaluate the association between lipid profiles and outcomes after RP; 3. Outcomes must include at least one of positive surgical margin (PSM), lymph node involvement (LNI), Gleason score (GS) on surgical specimen ≥8, pT on surgical specimen ≥T3 and BCR; 4. Lipid profiles must include at least one of TC, LDL, HDL and TG; 5. Data must be presented in the fashion of odds ratio (OR) or hazard ratio (HR); 6. Literature must be published in English. The excluding criteria were: 1. Patients were treated RP and radiation therapy or chemotherapy at the same, or patients were treated with either RP or other treatments, but the data was not distinguishable; 2. Publication not evaluating the association between lipid profiles and outcomes post RP; 3. Other lipid profiles rather than TC, LDL, HDL or TG were used for analysis; 4. Other postoperative outcomes rather than PSM, LNI, GS on surgical specimen ≥8, pT on surgical specimen ≥T3 or BCR were assessed; 5. Data was not presented in the fashion of OR and HR; 5. Literature published in non-English language.

### Data analysis

Two investigators independently extracted data from the included articles and all the members of our team resolved the discrepancies by consensus. All the analyses were performed using Review Manager (version 5.3) or STATA (version 12.0).

The primary outcome was the association between each lipid subfraction and each postoperative aggressive pathological outcome. Pooled OR value for abnormal versus normal lipid levels was estimated. Cut-off values for abnormal serum levels were commonly defined as ≥200 mg/dl for TC, ≥130 mg/dl for LDL, ≤40 mg/dl for HDL and ≥ 150 mg/dl for TG according to guidelines [17]. The second outcome was the association between each lipid subfraction and BCR, which was defined as a single prostate-specific antigen (PSA) > 0.2 ng/ml, two consecutive concentrations at 0.2 ng/ml, or secondary treatment for detectable postoperative PSA [11]. Pooled HR value of lipid profiles for BCR was calculated. When a trial presented both univariate and multivariate OR/HR, the latter was extracted for analysis.

Outcomes were taken as significant when the *P* value for Z test was < 0.05 or no intersection between the middle line of the forest plot and the diamond indicating the pooled effect estimate (OR/HR) happened. Heterogeneity among trials was tested using both *I*^2^ test or *Q* test. An *I*^2^ > 50% or *Q* test reporting *P* values < 0.1 were considered to denote heterogeneity. Sensitivity analyses were performed through the exclusion of one or more studies suspected of causing heterogeneity. Quality assessment of included studies was performed by two independent reviewers using Newcastle–Ottawa Scale (NOS) [18] and publication bias were assessed using funnel plot. When the two reviewers encountered discrepancies in the outcomes, they resolved those through discussion.

## Result

### Description of included studies

As showed in PRISMA flowchart (Additional file 1: Figure S1, 236 publications were identified and 55 of them were full-text reviewed for eligibility. Eventually, 12 articles involving 11,108 patients met the inclusion criteria and were included in the present study [11–14, 19–26] (Table 1). Seven of those studies purely emphasized lipid profiles while five other studies focused on MetS and prognosis after RP. All of those studies were published between 2014 and 2018. The cohort size varied from 199 to 3662 with a median follow-up ranged from 14.8 months to 134.4 months. All participants in those studies underwent RP (open, laparoscopic or robot-assisted). Statin use percentage varied from none to 50.7% in eight studies. Cofactors were inconsistently adjusted in multivariate analysis in those original trials. But Age, body mass index (BMI), preoperative PSA, Gleason score and statin were generally adjusted in most selected trials.

**Table 1:** Characteristics of the included studies

Study	Year	Country	Design	Size	Operation	Median Follow-Up (Month)	Mean Age	Statin Use	Cofactors
Post	2011	USA	Retrospective	383	RP	49	60.9	/	Age, Race, Preoperative PSA, Gleason Score, Tumor Stage, Surgical Margin Status, Smoking, and Other Metabolic Syndrome Components
Allott	2014	USA	Retrospective	843	RP	74.3	60.4	427 (50.7%)	Age, Race, Pre-Operative PSA, Year of Surgery, BMI, Surgical Center, Statin Use, Pathological Gleason Score, Prostate Weight, Positive Surgical Margins, Extracapsular Extension, and Seminal Vesicle Invasion
Shiota	2014	Japan	Prospective	283	Open (27.2%); LRP (14.8%); RALP (58.0%)	14.8	65 (49–78) ^a^	48 (20.2%)	Age, Pre-Operative PSA, Clinical T Stage, Pathological Stage, Biopsy Gleason Score, Surgical Approach, Surgical Margin, Pathological Gleason Score, Alteration of Gleason Score, Perineural Invasion, Angiolymphatic Invasion, Lymph Node Status
Jeannette	2015	Puerto Rico	Retrospective	199	RP	/	58.82	0	Age, BMI
Zhang	2015	China	Retrospective	322	RP + PLD	/	68	14 (4.3%)	Age, BMI, Hypertension, Diabetes, Smoking Status, Statin Usage, Preoperative PSA, Biopsy Gleason Score, Clinical Stage
Kang	2015	China	Retrospective	663	RP	21	68 (62–72) ^a^	/	Biopsy Gleason Score, Pathological Gleason Score, Preoperative PSA, Pathological T Stage, Lymph Node Metastasis, Surgical Margin
Yoshio	2016	Japan	Retrospective	562	Open (56.2%); RALP (43.8%)	54	65.9	69 (12.3%)	Age, Pre-Operative PSA, BMI, Statin Use, Clinical T Stage, Gleason Score, Extracapsular Extension, Seminal Vesicle Invasion, Surgical Margin, Lympho-Vascular Invasion, Perineural Invasion, Lymph Node Metastases
Bhindi	2016	Canada	Prospective	1939	Open (76%); LRP (6.1%); RALP (17.9%)	31	61.5	611 (31.5%)	Age, Year Of RP, And Statin Use, Disease Parameters (PSA At Surgery, Final Grade, Pathological Stage, Surgical Margin Status), Surgical Approach of RP And Type of Nerve Sparing
Wettstein	2017	Switzerland	Prospective	371	RP	28	63	61 (16.4%)	Age, PSA, Extra-prostatic disease (> = pT3), High-risk disease (> = Gleason 8), Positive nodal status (pN1), Positive surgical margins, Statin use
Colicchia	2017	USA	Prospective	3662	Open (69.4%); RALP (30.4%)	102	/	/	Age at Surgery, Total Number of Positive Cores, Max % Of Tumor in The Core, Clinical Stage, And Log 2 Pre-Surgery PSA.
Lebdai	2017	France	Retrospective	567	Open (13.6%) LRP (6.2%); RALP (78.1%)	/	64 (45–79) ^a^	/	BMI, Abdominal Perimeter, Glycemia, High Blood Pressure
Rantaniemi	2018	Finland	Retrospective	1314	RP	134.4	/	467 (35.5%)	Age, Preoperative PSA Level, Pathological T Stage and Gleason Score, Use of Antihypertensive and Antidiabetic Medication, Non-Steroidal Anti-Inflammatory Drugs and Allopurinol, and Surgical Marginal Positivity

*RP* radical prostatectomy, *LRP* laparoscopic radical prostatectomy, *RALP* robot-assisted radical prostatectomy, *PLD* pelvic lymphadenectomy, *PSA* prostate-specific antigen, *BMI* body mass index; /: data not available; a = median age

### Postoperative pathological outcomes

**Fig. 1:**
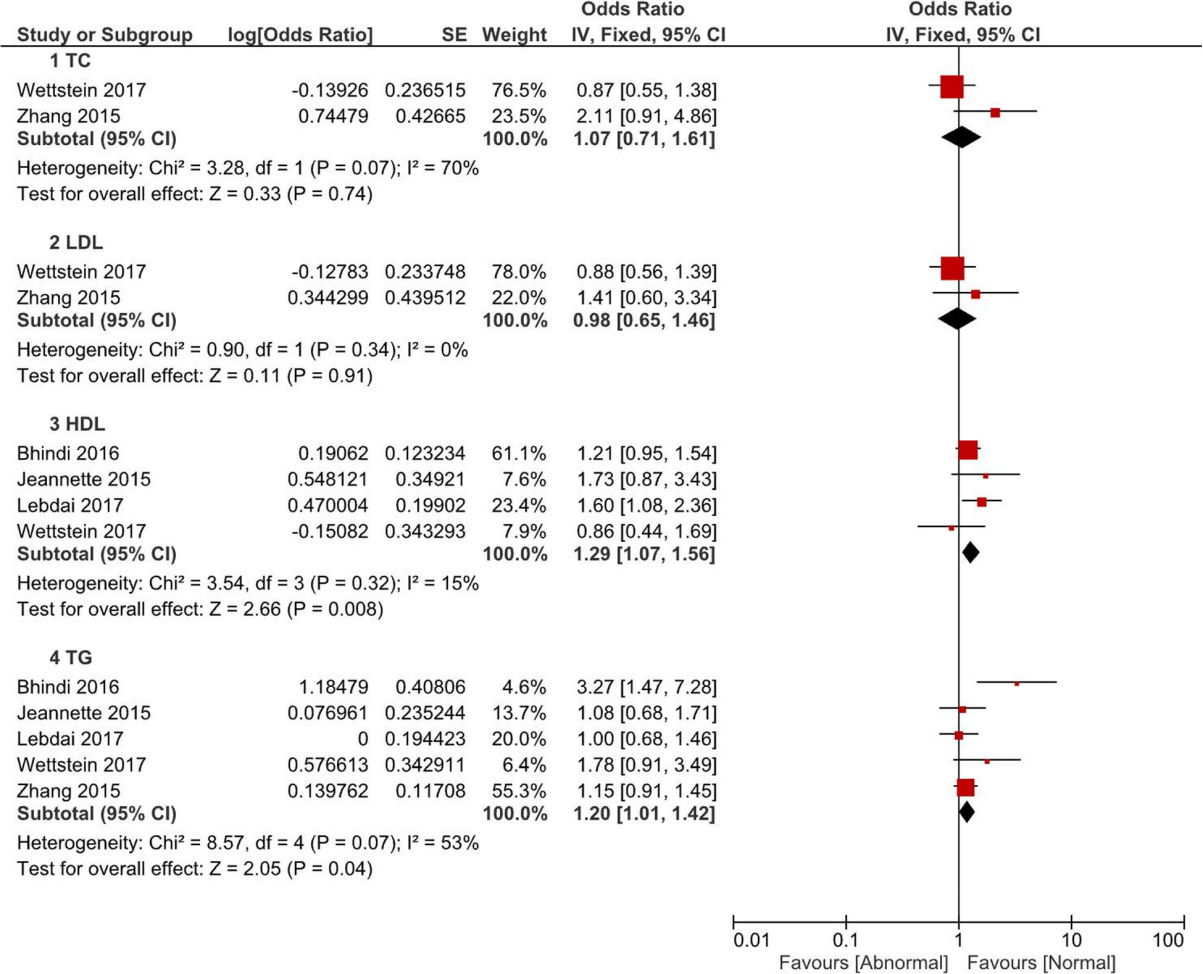
Pooled odd ratios of abnormal lipid profiles levels for pT ≥ T3 after radical prostatectomy

**Fig. 2:**
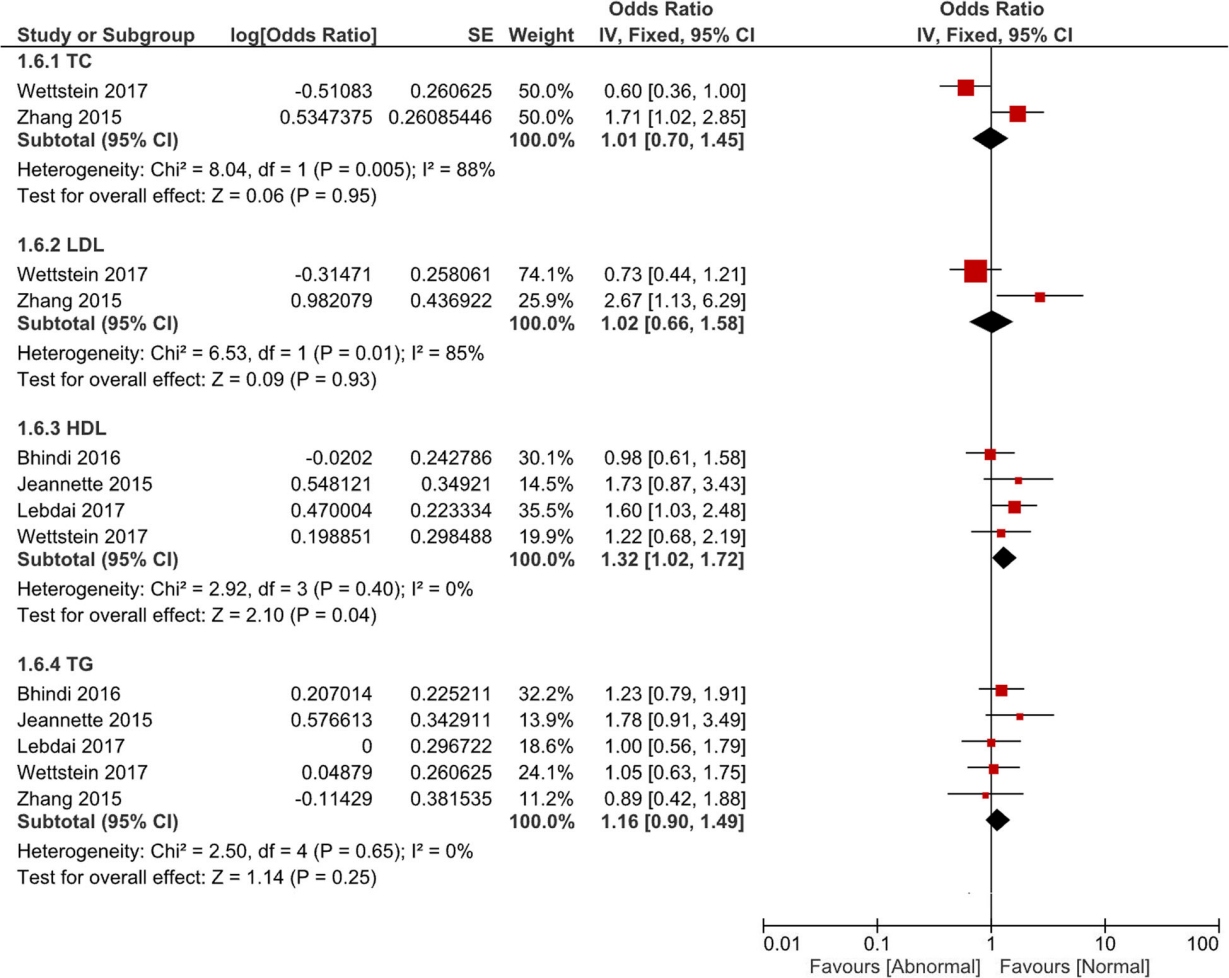
Pooled odd ratios of abnormal lipid profiles levels for GS ≥8 after radical prostatectomy

**Fig. 3:**
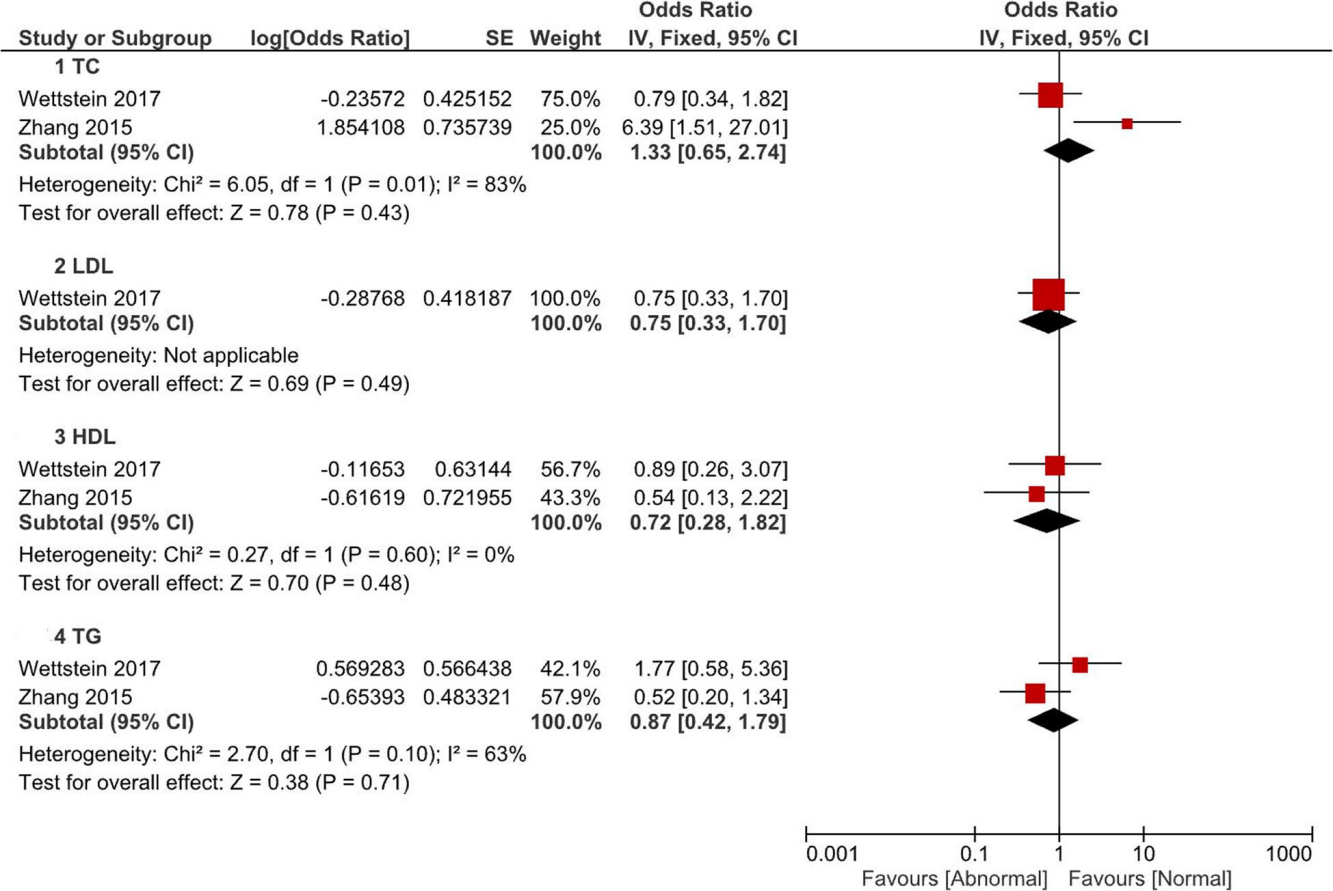
Pooled odd ratios of abnormal lipid profiles levels for LNI after radical prostatectomy

**Fig. 4:**
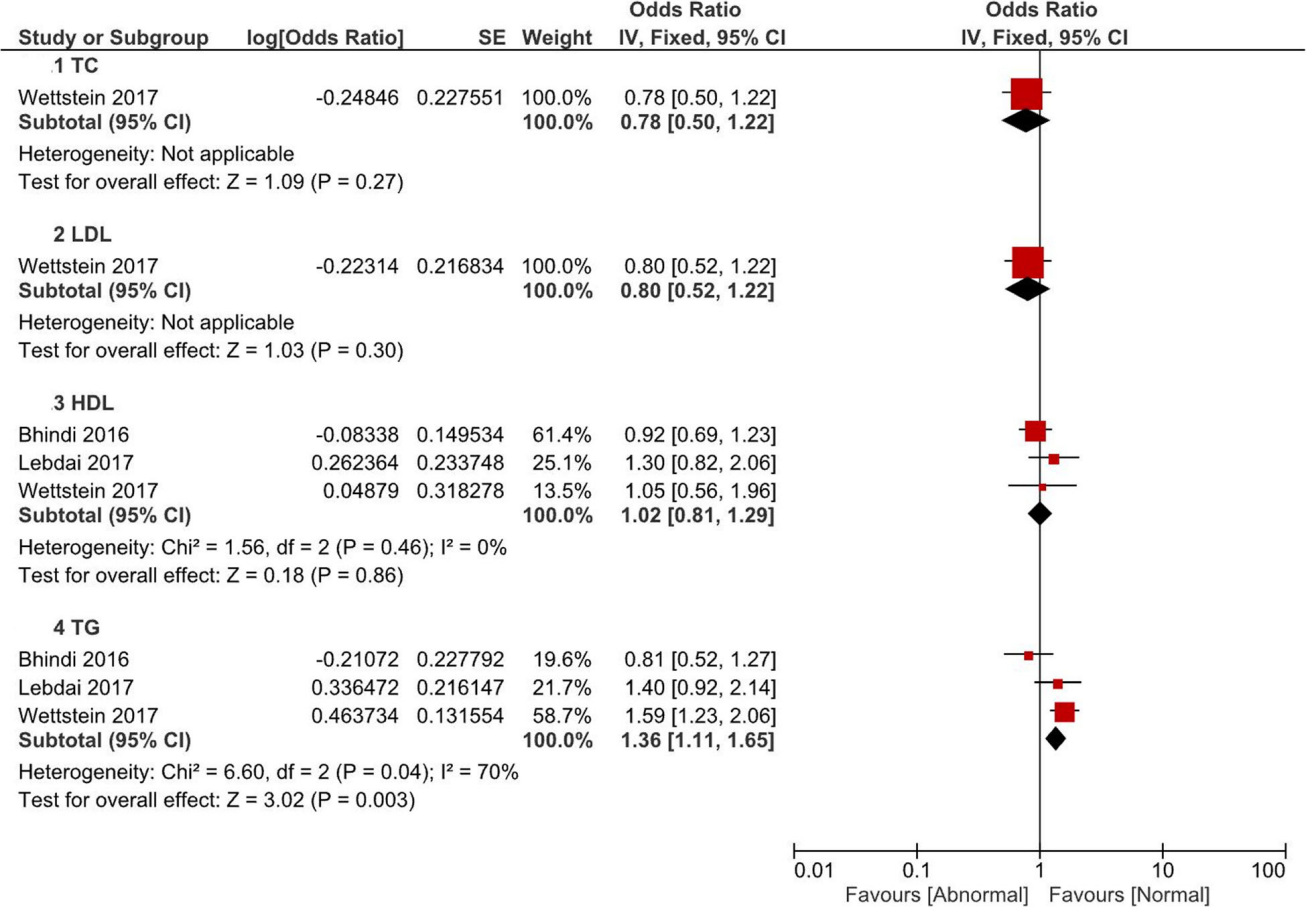
Pooled odd ratios of abnormal lipid profiles levels for PSM after radical prostatectomy

Comparisons of the occurrence of postoperative pathological outcomes between patients with and without abnormal baseline lipid levels were performed in the fashion of pooled OR value. All comparisons were grouped by TC, LDL, HDL and TG. In Fig. 1, patients with abnormal HDL (OR = 1.29, 95% CI 1.07–1.56, *P* = 0.008) or TG (OR = 1.20, 95% CI 1.01–1.42, *P* = 0.04) had a significant higher rate of pT ≥3. However, there was no significant difference of pT ≥3 associated with abnormal TC (*P* = 0.74) or LDL (*P* = 0.91). Postoperative pathological GS ≥8 was observed to be associated with abnormal HDL (OR = 1.32, 95% CI 1.02–1.72, *P* = 0.04) and TG (OR = 1.20, 95% CI 1.01–1.42, P = 0.04) (Fig. 2). Figure 3 and Fig. 4 showed that patients with abnormal lipid profile levels had generally similar risk of LNI and PSM. The only exception was that abnormal TG level was linked [12] with higher risk of PSM (OR = 1.36, 95% CI 1.11–1.65, *P* = 0.003).

### Biochemical recurrence

**Fig. 5:**
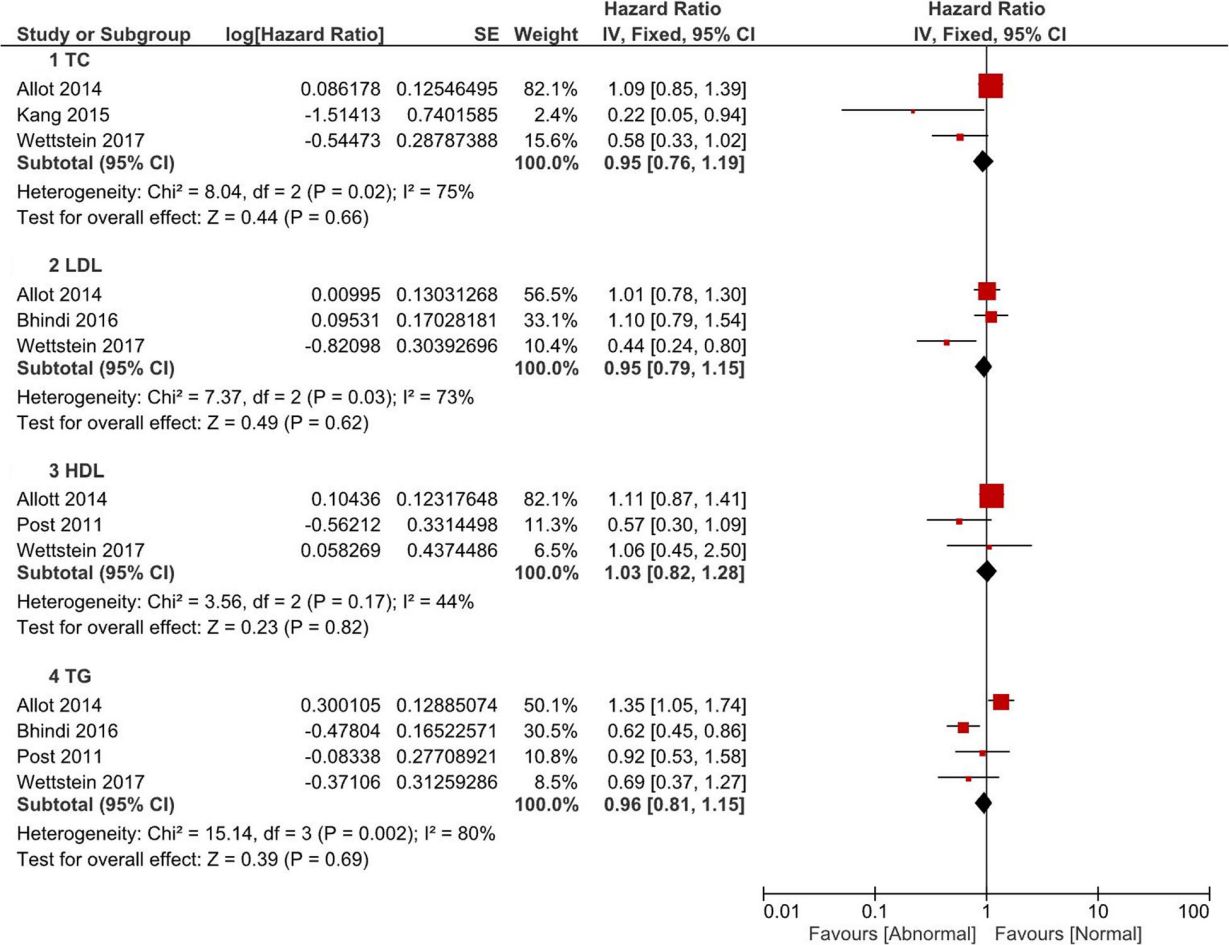
Pooled hazard ratios of abnormal lipid profiles levels for BCR after radical prostatectomy

Figure 5 displayed the adjusted pooled HR values of abnormal versus normal lipid profile levels for BCR. Several studies indicated lipid subfractions could lower the risk of BCR (adjusted HR = 0.22, 95% CI 0.05–0.94 of TC (Kang [12]), adjusted HR = 0.41, 95% CI 0.21–0.79 of HDL (Wettstein [13]), and adjusted HR = 0.62, 95% CI 0.45–0.86 of TG (Bhindi [22])). However, the pooled HR showed that, compared to normal levels, abnormal lipid profile levels led to a generally similar risk of BCR (TC: *P* = 0.66; LDL: *P* = 0.62; HDL: *P* = 0.50; TG: *P* = 0.69).

### Quality assessment and publication bias

The NOS for non-randomized studies was employed to evaluate the quality of the data and the risk of bias. The selection, compatibility, and outcome of those studies were assessed. Each study meeting one of the numbered items was awarded one star, with a possible maximum of nine stars awarded to a single study. Studies awarded at least seven stars were defined as high quality. Additional file 1: Table S1 summarized the outcomes of quality assessment. Accordingly, all of the included studies were of a relatively high quality. Funnel plots were also presented in supplementary materials and no evident publication bias were observed  (Figure S2-S6).

## Discussion

The current meta-analysis demonstrated low HDL was associated with more frequent occurrence of pathological T stage (pT) ≥ T3 (OR = 1.29, 95% CI 1.07–1.56, *P* = 0.008) and Gleason score (GS) ≥8 (OR = 1.32, 95% CI 1.02–1.72, *P* = 0.04) after RP. Hypertriglyceridemia was also linked with higher risk of pT ≥ T3 (OR = 1.20, 95% CI 1.01–1.42, P = 0.04) and positive surgical margin (PSM) (OR = 1.36, 95% CI 1.11–1.65, P = 0.04). However, no significant association was observed between BCR and abnormal lipid profile levels. Despite that researchers have conducted multiples studies, the role of lipid profiles in PCa still remains unclear. A recent meta-analysis suggested that serum TC, HDL, LDL and TG may not be associated with risk of overall PCa or high-grade PCa [10]. But opposite argument that metabolic syndrome (MetS), of which hypertriglyceridemia and low HDL levels may also be components [27], was associated with risk of overall PCa, in particularly high-grade PCa was proposed by Gacci [28]. Moreover, they also claimed MetS was linked with advanced tumor features and BCR. While several original researches presented controversial outcomes, data regarding prognostic value of lipid profiles after RP remains limited. To our knowledge, the current comprehensive systematic review and meta-analysis is the first to evaluate data about the prognostic value of lipid profiles after RP.

A range of studies investigated the potential mechanisms behind lipid profiles and PCa. As an immunocompetent organ, the prostate gland contains lymphocytes, macrophage and granulocytes and is able to secret various cytokine, chemokine and growth factors. Hypotheses including inflammation, membrane organization and effects on cell proliferation have been introduced [29–33]. Zhuang [29] and Solomon [30] used a xenograft model and observed hypercholesterolemia could accelerate the prostatic tumor growth. Furthermore, Llaverias also argued that hypercholesterolemia could lead to increased prostatic tumor volume and progression and metastases [31]. Some experiments also proved the involvement of cholesterol in element controlling signaling events of PCa cells [29, 32, 33]. In summary, it is feasible to assume that faster growing PCa cells have higher consumption of cholesterol. Thus, our outcomes that abnormally high levels of cholesterol or TG promote more advanced pathologic tumors features after RP looks reasonable. A cohort study supporting this hypothesis by Schnoeller [34] defined patients with pT3–4 and/or pN+ and/or GS ≥8 after RP as high-risk and showed hypercholesterolemia was a risk factor of high-risk PCa (OR = 2.01, *P* < 0.001). Using similar settings, Zhao [35] reported low level of HDL was attributable to high-risk PCa. Not only that, abnormal LDL levels could also lead to higher risk of BCR, claimed by Macleod [36]. Even though, there is still a lack of definitive conclusion between lipid profiles and PCa pathogenesis. Therefore, high-quality translational research and randomized control trials are further needed.

Known as an important cholesterol-controlling medicine, statin was believed to have contrast effect to cholesterol on PCa and commonly used. A flaw of our study was that data of statin use at baseline was not complete and we could not factor statin use into meta-analysis. However, evidence indicated that statin use had no significant association with BCR after RP [21, 26]. Recent studies by Murtola, Wettstein and Zhang also confirmed statin use was not an independent risk factor of advanced pathologic features or BCR after RP [6, 13, 14]. Moreover, three meta-analyses supported these conclusion by demonstrating that statin use was not associated with either PCa risk [37] or BCR [38, 39]. What is more, a Lancet study even revealed there was no association between reduction of LDL by statin therapy with overall cancer incidence [40]. Therefore, it’s feasible to assume that the deficit of the data of statin use would not substantially affect our analysis.

For the first time, our study included 12 articles related to the prognostic value of lipid profiles after RP and perform quantitative analysis. Notably, to make our outcomes more reliable, we did not only include studies purely assessing lipid profiles, but also selected available MetS researches. However, our study should not interpret without limitation. First, baseline cofactors (age, BMI, race, PSA level, biopsy Gleason score and etc.) were major concerns that might influence our outcomes. Although instead of performing a crude analysis using the number of events of advanced pathological outcomes and BCR, we primarily extracted the adjusted OR and HR, but the cofactors adjusted in those studies were ununiformed, leading to a negative effect on the accuracy. Furthermore, this could also be the major reason causing the substantial heterogeneity in part of our analyses. Second, given that no related randomized controlled trials have been conducted, we systematically searched the mainstream database but could only include prospective and retrospective studies. It’s noted although these selected studies were not highest-level evidence, but all variables and outcomes were recorded pre- or after surgery rather than recalled by patients. Thus, recall bias could be avoided. Third, data of some important outcomes including overall mortality and cancer-specific mortality were not reported in those studies and we were also unable to perform further analysis.

On the basis of existed original studies, the aim of our study is to maximally discriminate the prognostic value of lipid profiles after RP. Our data should be carefully assessed in decision-making of treatment and follow-up. However, it is obvious that more high-quality researches, in particular randomized controlled trials and basic research, are warranted to verify our findings. Future studies should evaluate the prognostic value of lipid profile of not only pathologic outcomes but also oncologic outcomes. Also, considering that GS 3 + 4 and GS4 4 + 3 have been categorized differently, it would be meaningful for future studies to put more effort into this issue.

## Conclusion

In this meta-analysis, we found that low HDL level (≤40 mg/dl) was associated with pT ≥ T3 and GS ≥8 after RP, and we also found that elevated serum TG level (≥150 mg/dl) was linked with pT ≥ T3 and PSM. There was no significant correlation between other abnormal lipid subfractions levels with advanced pathologic features or BCR after RP.

## Take home message

1. Our meta-analysis is the first to evaluate the association between lipid profiles and prognosis after radical prostatectomy
2. Elevated TC level was associated with LNI and pT ≥ T3.
3. Elevated triglycerides level was linked with pT ≥ T3.
4. Lipid profiles were not correlated with biochemical recurrence.

## Additional file

Additional file 1: **Table S1.** Quality assessment of the included studies by Newcastle-Ottawa Scale. **Figure S1.** PRISMA flowchart of literature selection. **Figure S2.** Publication bias of data for lipid profiles and pathological T stage ≥T3 after radical prostatectomy. **Figure S3.** Publication bias of data for lipid profiles and Gleason score ≥ 8 after radical prostatectomy. **Figure S4.** Publication bias of data for lipid profiles and lymph node involvement after radical prostatectomy. **Figure S5.** Publication bias of data for lipid profiles and positive surgical margin after radical prostatectomy. **Figure S6.** Publication bias of data for lipid profiles and biochemical recurrence after radical prostatectomy. (DOCX 227 kb)

## Abbreviations

BCR: Biochemical recurrence
BMI: Body mass index
GS: Gleason score
HDL: High-density lipoprotein
HR: Hazard ratio
LDL: Low-density lipoprotein
LNI: Lymph node involvement
MetS: Metabolic syndrome
NOS: Newcastle-Ottawa scale
OR: Odds ratio
PCa: Prostate cancer
PSA: Prostate-specific antigen
PSM: Positive surgical margin
RP: Radical prostatectomy
TC: Total cholesterol
TG: Triglycerides
