# Supplementary material for: Prognostic value of lipid profiles after radical prostatectomy: a systematic review and meta-analysis
Source: Lipids Health Dis. 2019 May 28;18:124. doi: 10.1186/s12944-019-1068-6 (PMC6540553; doi:10.1186/s12944-019-1068-6)

**Table S1.** Quality assessment of the included studies by Newcastle-Ottawa Scale

| Study | Year | Selection | | | | comparability | | Exposure | | | No. of Stars |
| --- | --- | --- | --- | --- | --- | --- | --- | --- | --- | --- | --- |
|  |  | REC | SNEC | AE | DO | SC | AF | AO | FU | AFU |  |
| Post | 2015 | * | * | * | * | * | / | * | * | * | 8 |
| Allot | 2014 | * | * | * | * | / | * | * | * | * | 8 |
| Shiota | 2014 | * | * | * | * | * | * | * | * | * | 9 |
| Jeannett | 2015 | * | * | * | * | * | * | * | / | * | 8 |
| Zhang | 2015 | * | * | * | * | * | * | * | / | * | 8 |
| Kang | 2015 | * | * | * | * | * | / | * | * | * | 8 |
| Yoshio | 2016 | * | * | * | * | * | * | * | * | * | 9 |
| Bhindi | 2016 | * | * | * | * | * | * | * | * | * | 9 |
| Wettstein | 2017 | * | * | * | * | * | * | * | * | * | 9 |
| Colicchia | 2017 | * | * | * | * | / | / | * | * | * | 7 |
| Lebdai | 2017 | * | * | * | * | * | * | * | * | * | 9 |
| Rantaniemi | 2018 | * | * | * | * | * | / | * | * | * | 8 |

REC= representativeness of the cohort, SNEC= selection of the none posed cohort, AE= ascertainment of exposure, DO= demonstration that outcome of interest was not present at start of study, SC= study controls most important factors such as age and PSA level, AF= study controls for other important factors, AO= assessment of outcome, FU= follow-up long enough for outcomes to occur ('long enough' is defined as 1 year), AFU=adequacy of follow-up of cohort (≥ 80%). "*" means that the study is satisfied the item, and "/" means not.

**Figure S1.** PRISMA flowchart of literature selection


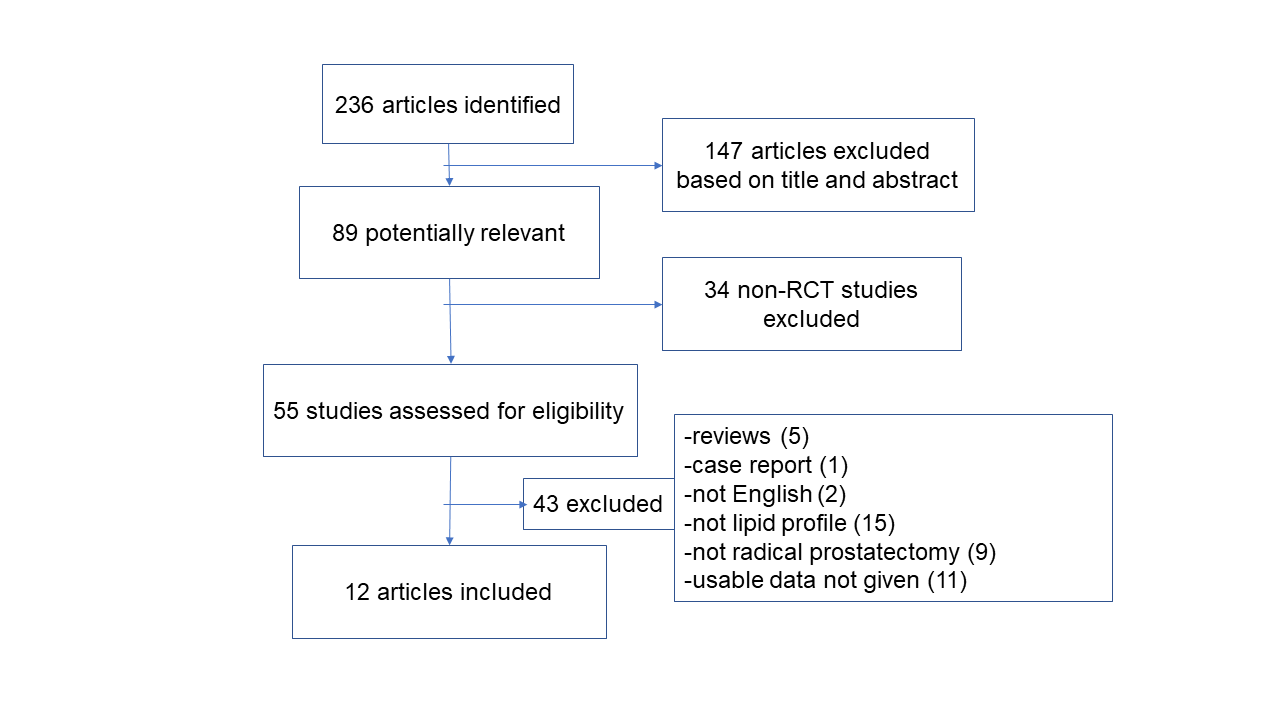


**Figure S2**. Publication bias of data for lipid profiles and pathological T stage ≥T3 after radical prostatectomy.


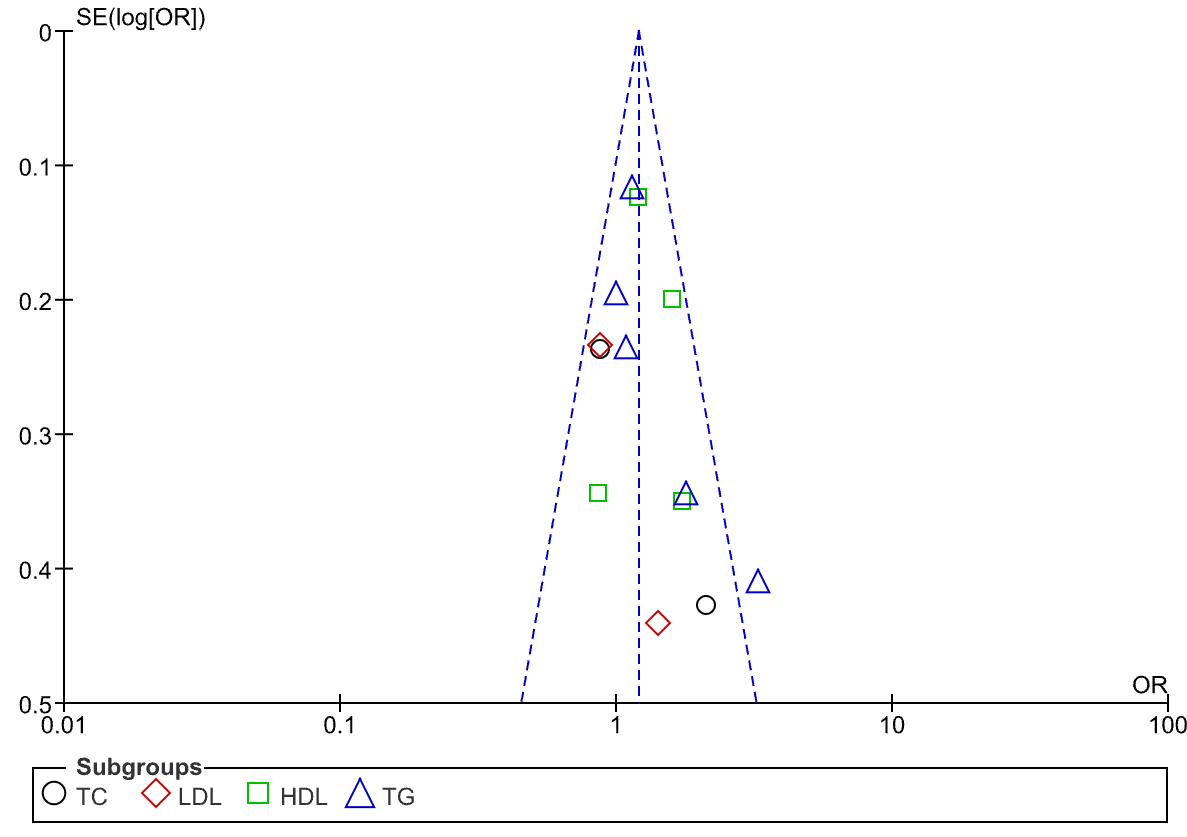


**Figure S3**. Publication bias of data for lipid profiles and Gleason score≥8 after radical prostatectomy.


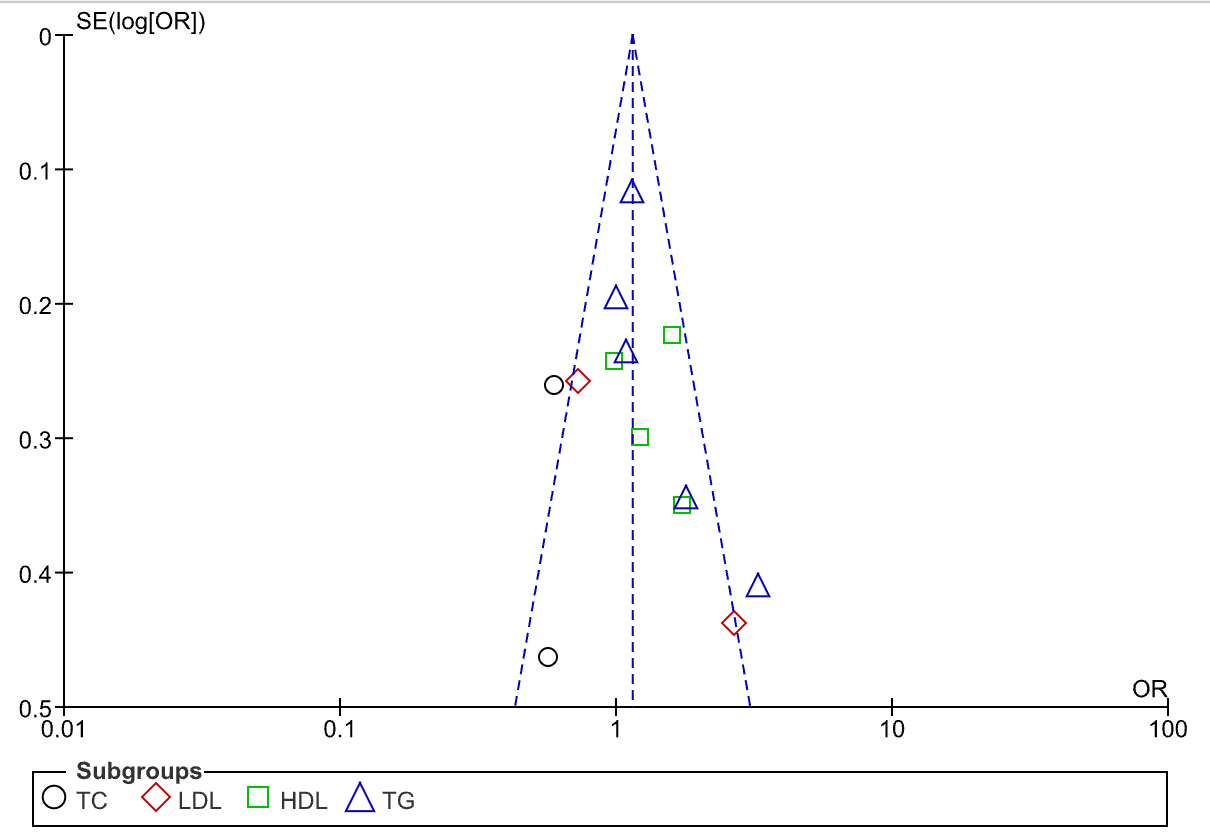


**Figure S4**. Publication bias of data for lipid profiles and lymph node involvement after radical prostatectomy.


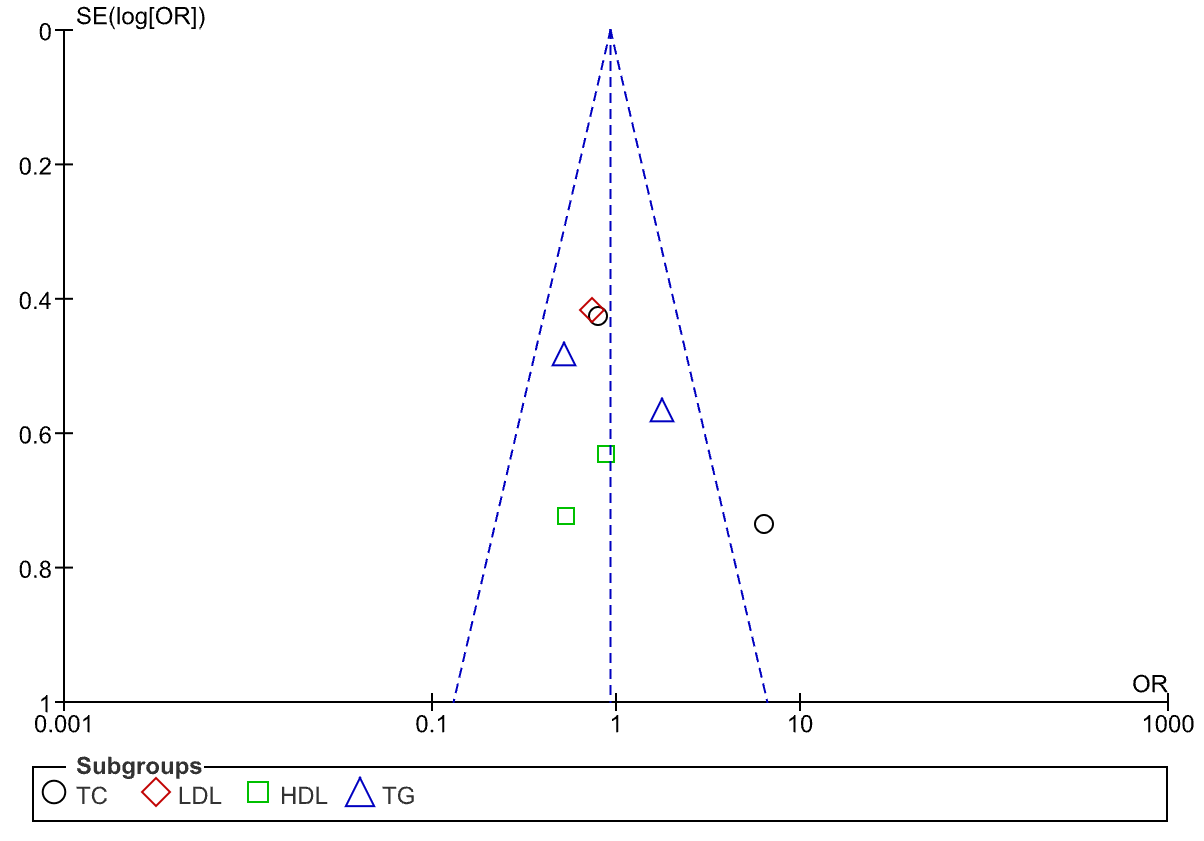


**Figure S5**. Publication bias of data for lipid profiles and positive surgical margin after radical prostatectomy


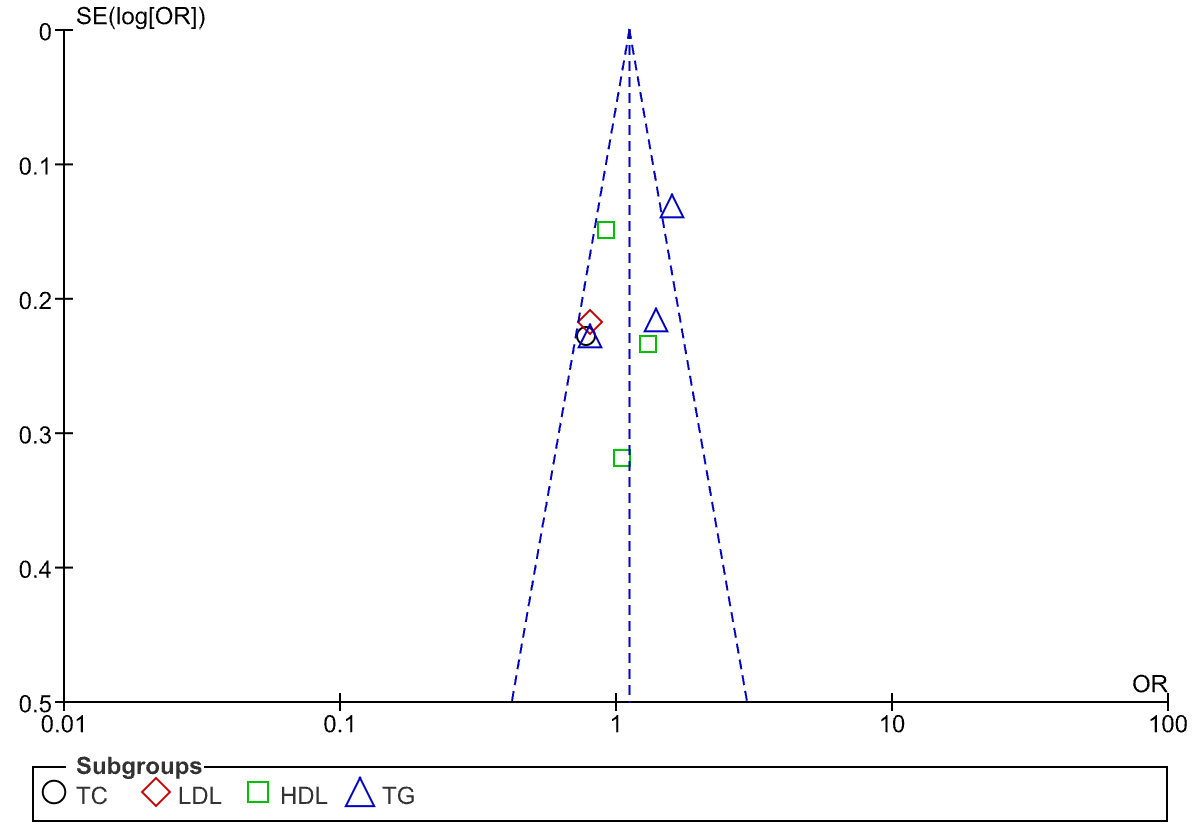


**Figure S6**. Publication bias of data for lipid profiles and biochemical recurrence after radical prostatectomy


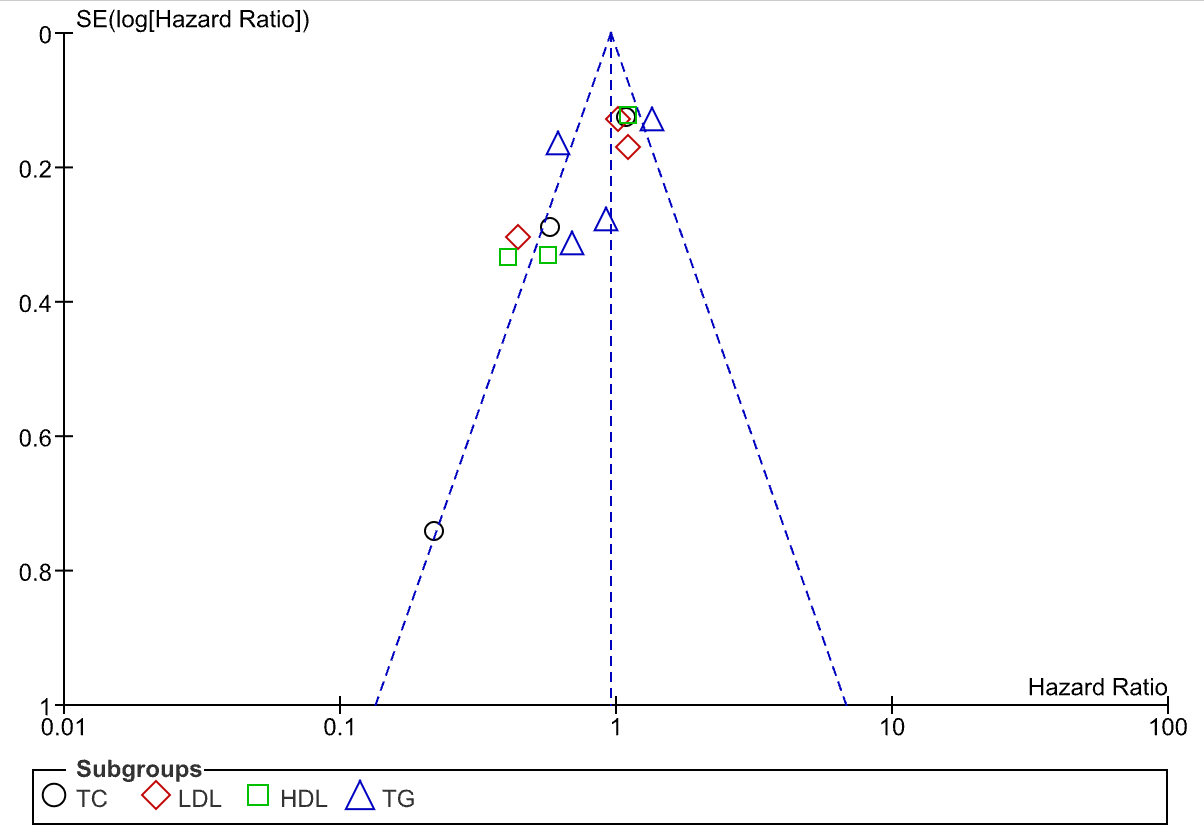

Supplement: Supplementary file 1 — Table S1. Quality assessment of the included studies by Newcastle-Ottawa Scale. Figure S1. PRISMA flowchart of literature selection. Figure S2. Publication bias of data for lipid profiles and pathological T stage ≥T3 after radical prostatectomy. Figure S3. Publication bias of data for lipid profiles and Gleason score ≥ 8 after radical prostatectomy. Figure S4. Publication bias of data for lipid profiles and lymph node involvement after radical prostatectomy. Figure S5. Publication bias of data for lipid profiles and positive surgical margin after radical prostatectomy. Figure S6. Publication bias of data for lipid profiles and biochemical recurrence after radical prostatectomy. (DOCX 227 kb) [file 12944_2019_1068_MOESM1_ESM.docx]
